# Supplementary material for: Photosynthetic responses of Halimeda scabra (Chlorophyta, Bryopsidales) to interactive effects of temperature, pH, and nutrients and its carbon pathways
Source: PeerJ. 2021 Mar 5;9:e10958. doi: 10.7717/peerj.10958 (PMC7938779; doi:10.7717/peerj.10958)
Supplement: Supplemental Information 1 — Experiment design to test the interactive effects of temperature, pH, and nutrients on gross photosynthesis of H. scabra. [file peerj-09-10958-s001.docx]

Table 1 S1:

Experiment design to test the interactive effects of temperature, pH, and nutrients on gross photosynthesis.

| Nutrients concentrations  (KNO_3_:K_3_PO_4_) | pH | Temperature (°C) |
| --- | --- | --- |
|  |  | 24°C |
|  | 7.5 | 28°C |
|  |  | 33°C |
|  |  | 24°C |
| 1.0:0.1 | 8.2 | 28°C |
|  |  | 33°C |
|  |  | 24°C |
|  | 8.6 | 28°C |
|  |  | 33°C |
|  |  | 24°C |
|  | 7.5 | 28°C |
|  |  | 33°C |
|  |  | 24°C |
| 5.0:0.5 | 8.2 | 28°C |
|  |  | 33°C |
|  |  | 24°C |
|  | 8.6 | 28°C |
|  |  | 33°C |
|  |  | 24°C |
|  | 7.5 | 28°C |
|  |  | 33°C |
|  |  | 24°C |
| 10.0:1.0 | 8.2 | 28°C |
|  |  | 33°C |
|  |  | 24°C |
|  | 8.6 | 28°C |
|  |  | 33°C |
|  | 7.5 | 24°C |
|  |  | 28°C |
|  |  | 33°C |
| Control treatment | 8.2 | 24°C |
|  |  | 28°C |
|  |  | 33°C |
|  | 8.6 | 24°C |
|  |  | 28°C |
|  |  | 33°C |
