# Supplementary material for: Photosynthetic responses of Halimeda scabra (Chlorophyta, Bryopsidales) to interactive effects of temperature, pH, and nutrients and its carbon pathways
Source: PeerJ. 2021 Mar 5;9:e10958. doi: 10.7717/peerj.10958 (PMC7938779; doi:10.7717/peerj.10958)
Supplement: Supplemental Information 2 — Results of three-way ANOVA and Post hoc Newman-Keuls. [file peerj-09-10958-s002.docx]

Table S2:

Results of three-way ANOVA and Post hoc Newman-Keuls.

| Sources of variation | mean | SS | DF | MS | F | *p* |
| --- | --- | --- | --- | --- | --- | --- |
| Temperature | | 10.032 | 2 | 5.016 | 45.30 | 0.000000 |
| 24°C | 1.1665^b^ |  |  |  |  |  |
| 28°C | 1.2219^b^ |  |  |  |  |  |
| 33°C | 1.4318^a^ |  |  |  |  |  |
| pH | | 0.629 | 2 | 0.315 | 2.84 | 0.058977ns |
| Nutrients  (KNO3:K3PO4) | | 1.584 | 3 | 0.528 | 4.77 | 0.002681 |
| 1.0:0.1 | 1.3085^a^ |  |  |  |  |  |
| 5.0:0.5 | 1.1977^b^ |  |  |  |  |  |
| 10.0:1.0 | 1.2794^a^ |  |  |  |  |  |
| 0.0:0.0  control | 1.3106^a^ |  |  |  |  |  |
| Temperature*pH | | 1.195 | 4 | 0.299 | 2.70 | 0.029806 |
| 24°C*pH 7.5 | 1.256^b^ |  |  |  |  |  |
| 24°C*pH 8.2 | 1.184^bc^ |  |  |  |  |  |
| 24°C*pH 8.6 | 1.059^c^ |  |  |  |  |  |
| 28°C*pH 7.5 | 1.197^bc^ |  |  |  |  |  |
| 28°C*pH 8.2 | 1.256^b^ |  |  |  |  |  |
| 28°C*pH 8.6 | 1.213^bc^ |  |  |  |  |  |
| 33°C*pH 7.5 | 1.435^a^ |  |  |  |  |  |
| 33°C*pH 8.2 | 1.438^a^ |  |  |  |  |  |
| 33°C*pH 8.6 | 1.427^a^ |  |  |  |  |  |
| Temperature*nutrients | | 6.858 | 6 | 1.143 | 10.32 | 0.000000 |
| 24°C*1.0:0.1 | 1.408^a^ |  |  |  |  |  |
| 24°C*5.0:0.5 | 1.081^d^ |  |  |  |  |  |
| 24°C*10:1.0 | 1.094^d^ |  |  |  |  |  |
| 24°C*control | 1.082^d^ |  |  |  |  |  |
| 28°C*1.0:0.1 | 1.163^cd^ |  |  |  |  |  |
| 28°C*5.0:0.5 | 1.149^d^ |  |  |  |  |  |
| 28°C*10.0:1.0 | 1.204^bcd^ |  |  |  |  |  |
| 28°C*control | 1.372^ab^ |  |  |  |  |  |
| 33°C*1.0:0.1 | 1.355^abc^ |  |  |  |  |  |
| 33°C*5.0:0.5 | 1.363^ab^ |  |  |  |  |  |
| 33°C*10.0:1.0 | 1.540^a^ |  |  |  |  |  |
| 33°C*control | 1.478^a^ |  |  |  |  |  |
| pH*nutrients | | 6.130 | 6 | 1.022 | 9.23 | 0.000000 |
| 7.5*1.0:0.1 | 1.291^abc^ |  |  |  |  |  |
| 7.5*5.0:0.5 | 1.296^abc^ |  |  |  |  |  |
| 7.5*10.0:1.0 | 1.149^cd^ |  |  |  |  |  |
| 7.5*control | 1.446a |  |  |  |  |  |
| 8.2*1.0:0.1 | 1.349^ab^ |  |  |  |  |  |
| 8.2*5.0:0.5 | 1.202^bcd^ |  |  |  |  |  |
| 8.2*10.0:1.0 | 1.274^abcd^ |  |  |  |  |  |
| 8.2*control | 1.349^ab^ |  |  |  |  |  |
| 8.6*1.0:0.1 | 1.285^abcd^ |  |  |  |  |  |
| 8.6*5.0:0.5 | 1.095^d^ |  |  |  |  |  |
| 8.6*10.0:1.0 | 1.414^a^ |  |  |  |  |  |
| 8.6*control | 1.138^cd^ |  |  |  |  |  |
| Temperature*pH*nutrients | | 6.070 | 12 | 0.506 | 4.57 | 0.000000 |
| 24°C*7.5*1.0:0.1 | 1.406 ^bcde^ |  |  |  |  |  |
| 24°C*7.5*5.0:0.1 | 1.446^bc^ |  |  |  |  |  |
| 24°C*7.5*10.0:1.0 | 1.022^fgh^ |  |  |  |  |  |
| 24°C*7.5*control | 1.147^bcdefgh^ |  |  |  |  |  |
| 24°C*8.2*1.0:0.1 | 1.451^bc^ |  |  |  |  |  |
| 24°C*8.2*5.0:0.5 | 0.877^h^ |  |  |  |  |  |
| 24°C*8.2*10.0:1.0 | 1.196^bcdefgh^ |  |  |  |  |  |
| 24°C*8.2*control | 1.213^bcdefgh^ |  |  |  |  |  |
| 24°C*8.6*1.0:0.1 | 1.366^bcdef^ |  |  |  |  |  |
| 24°C*8.6*5.0:0.5 | 0.921^gh^ |  |  |  |  |  |
| 24°C*8.6*10.0:1.0 | 1.063^efgh^ |  |  |  |  |  |
| 24°C*8.6*control | 0.887^h^ |  |  |  |  |  |
| 28°C*7.5*1.0:0.1 | 1.186^bcdefgh^ |  |  |  |  |  |
| 28°C*7.5*5.0:0.1 | 1.105^bcdefgh^ |  |  |  |  |  |
| 28°C*7.5*10.0:1.0 | 1.081^cdefgh^ |  |  |  |  |  |
| 28°C*7.5*control | 1.413^bcde^ |  |  |  |  |  |
| 28°C*8.2*1.0:0.1 | 1.132^bcdefgh^ |  |  |  |  |  |
| 28°C*8.2*5.0:0.5 | 1.271^bcdefg^ |  |  |  |  |  |
| 28°C*8.2*10.0:1.0 | 1.179^bcdefgh^ |  |  |  |  |  |
| 28°C*8.2*control | 1.442^bcd^ |  |  |  |  |  |
| 28°C*8.6*1.0:0.1 | 1.169^bcdefgh^ |  |  |  |  |  |
| 28°C*8.6*5.0:0.5 | 1.069^defgh^ |  |  |  |  |  |
| 28°C*8.6*10.0:1.0 | 1.352^bcdef^ |  |  |  |  |  |
| 28°C*8.6*control | 1.260^bcdefg^ |  |  |  |  |  |
| 33°C*7.5*1.0:0.1 | 1.279^bcdef^ |  |  |  |  |  |
| 33°C*7.5*5.0:0.1 | 1.337^bcdef^ |  |  |  |  |  |
| 33°C*7.5*10.0:1.0 | 1.345^bcdef^ |  |  |  |  |  |
| 33°C*7.5*control | 1.779^a^ |  |  |  |  |  |
| 33°C*8.2*1.0:0.1 | 1.464^b^ |  |  |  |  |  |
| 33°C*8.2*5.0:0.5 | 1.457^bc^ |  |  |  |  |  |
| 33°C*8.2*10.0:1.0 | 1.447^bcd^ |  |  |  |  |  |
| 33°C*8.2*control | 1.385^bcdef^ |  |  |  |  |  |
| 33°C*8.6*1.0:0.1 | 1.320^bcdef^ |  |  |  |  |  |
| 33°C*8.6*5.0:0.5 | 1.294^bcdef^ |  |  |  |  |  |
| 33°C*8.6*10.0:1.0 | 1.828^a^ |  |  |  |  |  |
| 33°C*8.6*control | 1.268^bcdefg^ |  |  |  |  |  |
